# Cryoablation Versus Breast-Conserving Surgery for Early-Stage, Low-Risk Breast Cancer ≤ 1.5 cm: A Cost-Effectiveness Analysis

**DOI:** 10.1007/s00270-025-04269-3

**Published:** 2025-11-18

**Authors:** Xiao Wu, Kanti Pallav Kolli, Rita A. Mukhtar, Maggie Chung, Bonnie N. Joe, Ryan M. Kohlbrenner

**Affiliations:** 1 Interventional Radiology, Department of Radiology and Biomedical Imaging, University of California San Francisco, 505 Parnassus Ave, M361, San Francisco, CA 94143 USA; 2 Department of Surgery, University of California San Francisco, San Francisco, CA USA; 3 Breast Imaging, Department of Radiology and Biomedical Imaging, University of California San Francisco, San Francisco, CA USA

**Keywords:** Breast cryoablation, Breast conserving surgery, Early-stage breast cancer, Cost-effectiveness analysis

## Abstract

**Purpose:**

To compare ultrasound-guided cryoablation (BCA) and breast-conserving surgery (BCS) for patients with early-stage, low-risk breast cancer using a cost-effectiveness analysis.

**Materials and Methods:**

A Markov decision tree was constructed comparing BCA and BCS for unifocal small (≤ 1.5 cm), lymph node-negative, ultrasound-visible breast cancer from a payer’s perspective over a 5-year horizon. Clinical outcomes after cryoablation were based on the ICE3 trial, and those after BCS were based on a meta-analysis of 17 trials. Outcomes were measured in US dollars and quality-adjusted life years (QALY). Base case calculation, probabilistic and deterministic sensitivity analyses were performed.

**Results:**

Base case analysis showed BCA achieved comparable health outcomes (0.01–0.09 QALY higher) at a lower cost (at least $17,682 of cost saving per patient). Probabilistic sensitivity analysis showed cryoablation to be the better strategy in majority of the iterations driven by its lower procedural cost. BCS became the better strategy when the annual mortality after BCA was > 2.1%, equivalent to a 5-year cancer survival after BCA < 90.0%. BCA was the more optimal strategy when its annual local recurrence risk was < 51.5% or distant recurrence risk was < 1.04%. BCS became the more cost-effective strategy if the cost of BCA was $20,906 more than BCS. BCA remained more cost-effective when accounting for follow-up, provided the difference in costs between BCA and BCS was less than $4000 annually.

**Conclusion:**

BCA is a cost-effective strategy for patients with early-stage, low-risk, sonographically visible breast cancer when compared to BCS.

**Graphical Abstract:**

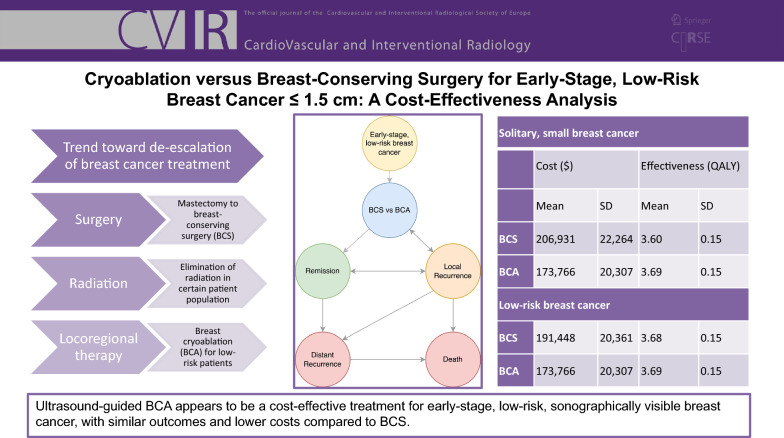

**Supplementary Information:**

The online version contains supplementary material available at 10.1007/s00270-025-04269-3.

## Introduction

Breast cancer is the most common type of cancer and the second leading cause of cancer-related death among women in the United States. Approximately 12.3% of U.S. women will be diagnosed with breast cancer during their lifetimes [1]. Over half of newly diagnosed breast cancers are early-stage at diagnosis [2]. With increased understanding of the disease, there has been a trend toward treatment de-escalation in patients with low-risk features [3]. Since the introduction of breast-conserving surgery (BCS) in the 1980s, BCS in combination with radiation therapy has become the standard of care for early-stage cancers given comparable outcomes with mastectomy [4]. However, BCS is often performed under general anesthesia, which can pose increased risks for patients with significant cardiopulmonary comorbidities [5].

Percutaneous microwave, radiofrequency, cryo- and laser ablation have been explored as minimally invasive surgical alternatives in some breast cancer patients. The concept of breast cryoablation (BCA) was first explored in 1985 using a mouse model [6]. It was approved by the Food and Drug Association in 2002 for symptomatic fibroadenomas, demonstrating high technical success, patient satisfaction, and volume reduction rates [7]. BCA for malignant lesions has been explored in several prospective and retrospective studies, especially after showing axillary lymph node dissection can be omitted [8]. Prospective 5-year follow-up data from the recently published ICE3 trial showed that BCA was safe and effective for small, ultrasound (US)-visible breast cancers [9]. These favorable oncologic outcomes further support the use of BCA, which has been shown to be associated with shorter procedure times, better tolerability, and favorable aesthetic outcomes compared to surgery [10]. The purpose of this study is to perform a cost-effectiveness analysis comparing BCS and BCA for early-stage, low-risk breast cancer.

## Methods

Approval from the institutional review board was not required as no patient-level data was used. The study follows Consolidated Health Economic Evaluation Reporting Standards (CHEERS) [11].

A modeling-based cost-effectiveness analysis was performed comparing BCS and BCA for patients with early-stage, low-risk invasive ductal carcinoma of the breast from a healthcare payer’s perspective. The inclusion criteria for the study are patients with a single, small (≤ 1.5 cm), node-negative, ultrasound-visible breast cancer. Low risk is defined as cancers that are positive for hormonal receptors and negative for HER2, with a histology grade of low to intermediate. The model was created with TreeAge Pro Healthcare 2024 (Cambridge, MA, USA), with a cycle length of 1 year.

### Model Design

**Fig. 1:**
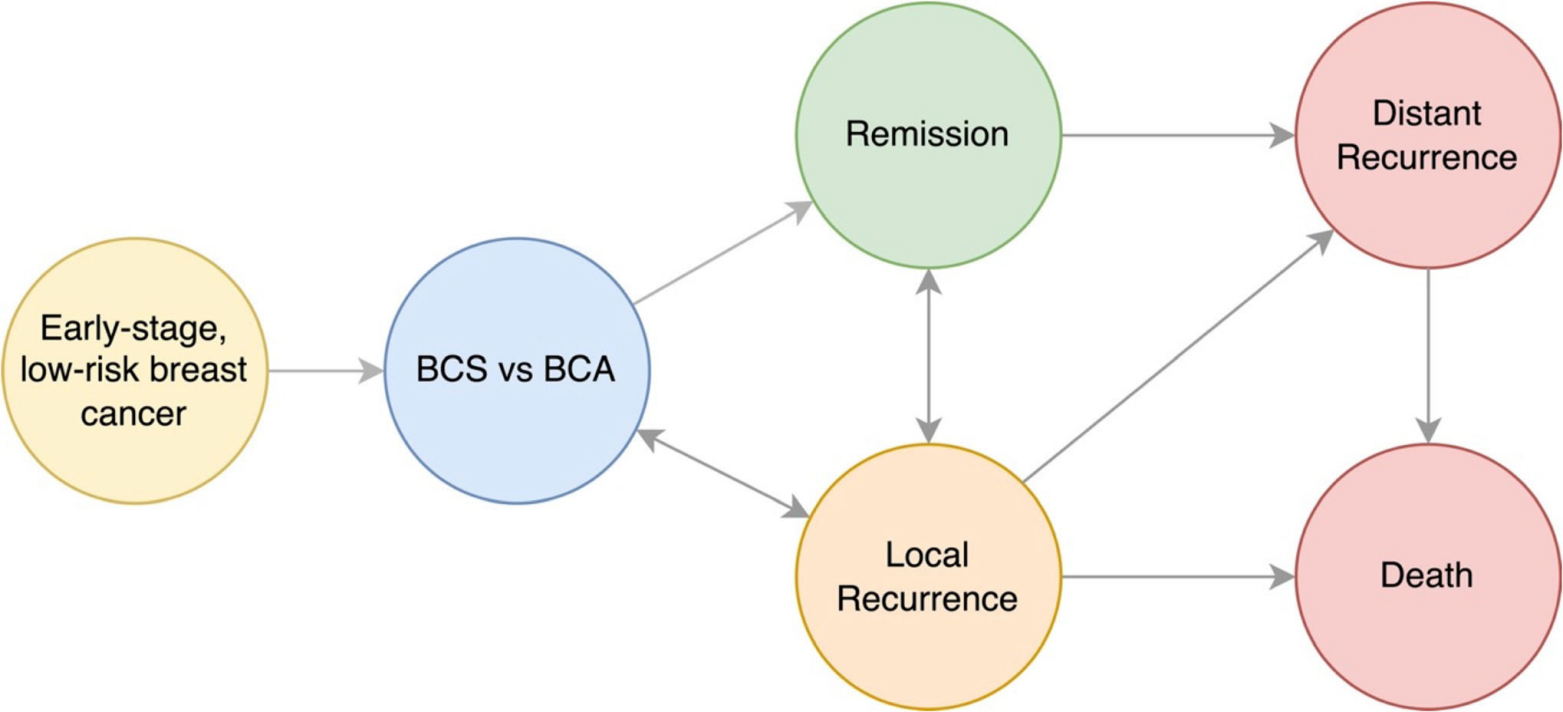
State transition diagram of the Markov model

The model started with a patient with a solitary, early-stage, low-risk breast cancer undergoing treatment, either BCS or BCA. Treatment-related complications were incorporated into the model. After the treatment, patients received clinical and imaging surveillance. Some received adjuvant therapy. Both local and distant recurrence was considered, with associated cost and disutility. Breast cancer specific mortality was also incorporated into the model. De novo second primary breast cancers were not included in the model, since the risk is independent of treatment modality. A total of 5 years follow-up or 5 Markov cycles were run. The Markov state transition diagram of the model is presented in Fig. 1.

### Model Parameters

Clinical outcomes after BCA were based on the ICE3 trial, a prospective, multi-center, single-arm trial involving 194 patients ≥ 60 years old. A total of 153 patients received adjuvant therapy at the discretion of treating physicians, including endocrine therapy only, radiation only, or the combination of both. Overall ipsilateral breast tumor recurrence (IBTR) was observed in 7 patients at a mean follow-up of 54 months. The cumulative IBTR at 5 years was 4.3% by Kaplan Meier analysis and breast cancer survival rate was 96.7% [9]. The outcomes after BCS were based on a meta-analysis of 17 trials by the Early Breast Cancer Trialists' Collaborative Group (EBCTCG) after BCS and radiotherapy. The pooled breast cancer mortality for patients with breast cancer ≤ 2 cm was 8.0% at 5 years and for patients with low-risk breast cancer was 2.9% [12]. All clinical parameters with their respective distributions are presented in Table 1.

**Table 1:** List of clinical parameters and their respective distributions

	Breast cryoablation		Breast conserving surgery	
	Mean	Distribution	Mean	Distribution
Procedural complication	Moderate: Wound complications/skin thermal injury: 2.4% [19]	Beta (5, 201)	Moderate: Wound complication:1.5% Hematoma/seroma: 11.0% Infection: 1.82% [26, 27]	Beta (266, 17,284) + Beta (1592, 15,958) + Beta (418, 22,583)
Procedural mortality	0		0	
Risk of total recurrence	5.3% at 5 years [9]		Low Risk: 1.6% over 5 years 1–20 mm: 8.0% over 5 years [12]	Low Risk: Beta (235, 2707) at 5 years (12) 1–20 mm: Beta (469, 5393)
Risk of annual local recurrence	0.86% [9]	Beta (8, 186) at 5 years	0.64% [12]	See above
Risk of distant recurrence	0.23% [9]	Beta (2, 192) at 54 months	0.96% [12]	See above
Breast cancer mortality at 5 years	0.73% [9]	Beta (7, 187) at 5 years	Low risk: Mortality 0.2% per year 1–20 mm: Mortality 0.91% per year [12]	Low risk: Beta (23, 9702) pear year 1–20 mm: Beta (265, 28,781) per year

Procedural costs of both BCS and BCA as well as breast cancer-related quality of life estimates were derived from Khan et al., who performed a prospective, longitudinal study involving patients with early, low-risk breast cancer undergoing BCS or BCA. Both direct and indirect costs were tabulated, and quality of life was assessed using the BREAST-Q score [13]. Subsequent medical costs associated with early- and advanced-stage disease (in the cases of distant metastasis) were derived from a SEER Medicare database analysis, which also accounted for adjuvant therapies such as systemic therapy and radiation [14]. All cost and utility parameters with their respective distributions are presented in Table 2.

**Table 2:** List of cost and utility parameters with their respective distributions

Costs	Breast cryoablation		Breast conserving surgery	
	Mean	Distribution	Mean	Distribution
Procedural cost	$2,501 [13]	Gamma with SD of $500	$18,859 [13]	Gamma with SD of $3,772
Cost of complications	$817 [26]	Gamma with SD of $39	$817 [26]	Gamma with SD of $39
Cost of routine medical cost for stage I disease	$36,351 [14]	Gamma with SD of 4401	$36,351 [14	Gamma with SD of 4401
Cost of local recurrence	$36,351 [14]	Gamma with SD of 4401	$36,351 [14]	Gamma with SD of 4401
Cost of distant recurrence and advanced disease	$153,049 [14]	Gamma with SD of 54,514	$153,049 [14]	Gamma with SD of 54,514
*Utilities*				
Disutility associated with treatment	−0.02 [13]	None	−0.10 [13]	None
Disutility associated with complications	−0.05*	None	−0.05*	None
Quality of life 1st year after primary breast cancer	0.696 [28]	Normal, SD = 0.029	0.696 [28]	Normal, SD = 0.029
Quality of life subsequent year after primary breast cancer	0.779 [28]	Normal, SD = 0.038	0.779 [28]	Normal, SD = 0.038
Quality of life with local recurrence	0.779 [28]	Normal, SD = 0.038	0.779 [28]	Normal, SD = 0.038
Quality of life with distant recurrence	0.685 [28]	Normal, SD = 0.029	0.685 [28]	Normal, SD = 0.029
Quality of life with mortality	0**		0**	

*By assumption

**Reference value

### Statistical Analysis

Two sets of base case calculations were performed using the most probable value of each parameter, assuming outcomes for patients with 1–20 mm breast cancer and with low-risk breast cancer. A willingness-to-pay (WTP) threshold was set at $100,000/quality-adjusted life year (QALY), and a 3% annual discount rate was used per standard cost-effectiveness analysis protocol in the United States [15, 16]. Separate subgroup analyses were performed using a WTP of $50,000/QALY. A dominant strategy was defined as one with lower cost and higher effectiveness. If a strategy had higher cost and effectiveness relative to the reference strategy, the incremental cost-effectiveness ratio (ICER), defined as incremental cost divided by incremental effectiveness, was compared against the WTP.

Probabilistic sensitivity analysis (Monte Carlo simulation) was performed by bootstrapping all input parameters from their distributions to simulate parallel cohorts of 10,000 BCS and BCA patients. Deterministic sensitivity analyses were performed by varying one or multiple variables over pre-defined range(s) while keeping the remaining variables at their mean value using net monetary benefit (NMB) as the metric. All sensitivity analyses were performed using outcomes associated with low-risk breast cancer in the source paper, which during subgroup analyses were more favorable than using 2 cm as a size cutoff [12].

## Results

Base case analysis after BCS for 1–20 mm and low-risk breast cancers are presented in Table 3. Both sets showed BCA to achieve comparable health benefit at a lower cost. The difference in quality of life was 0.01–0.09 QALY, equivalent to 4 to 33 days of life in perfect health. In terms of cost, BCA was approximately $17,000–$33,000 less expensive per patient in both scenarios.

**Table 3:** Base case calculation results

	Solitary, small breast cancer											Low-risk breast cancer			
	Cost ($)						Effectiveness (QALY)					Cost ($)		Effectiveness (QALY)	
	Mean				SD		Mean			SD		Mean	SD	Mean	SD
BCS	206,931				22,264		3.60			0.15		191,448	20,361	3.68	0.15
BCA	173,766				20,307		3.69			0.15		173,766	20,307	3.69	0.15

**Fig. 2:**
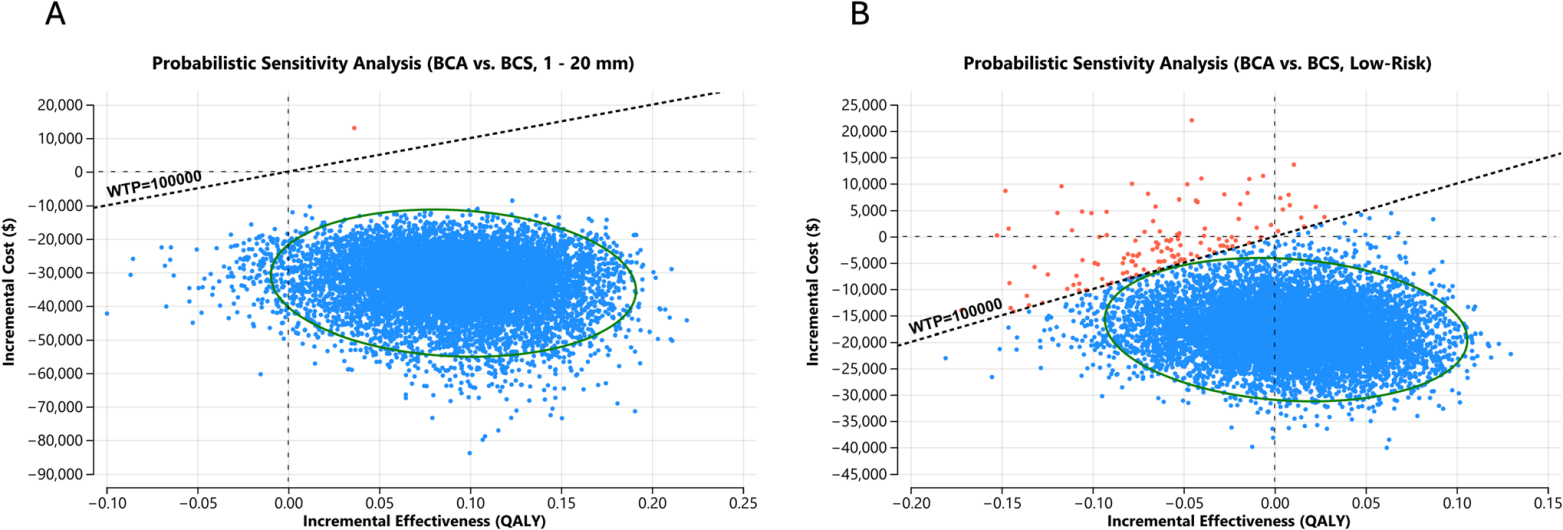
Probabilistic sensitivity analysis comparing BCA and BCS in patients with **A** breast cancer 0–20 mm and **B** low-risk breast cancer

Probabilistic sensitivity analysis with 10,000 iterations showed BCA to be the better strategy in 99–100% of the iterations in both scenarios, primarily driven by lower procedural costs BCA with comparable immediate and long-term outcomes of both strategies (Fig. 2A and B).

Tornado diagrams showed that the costs of local recurrences, breast cancer mortality, and recurrence risks (both local and distant) had the greatest impact on the model outputs (Supplemental Fig. 1).

**Fig. 3:**
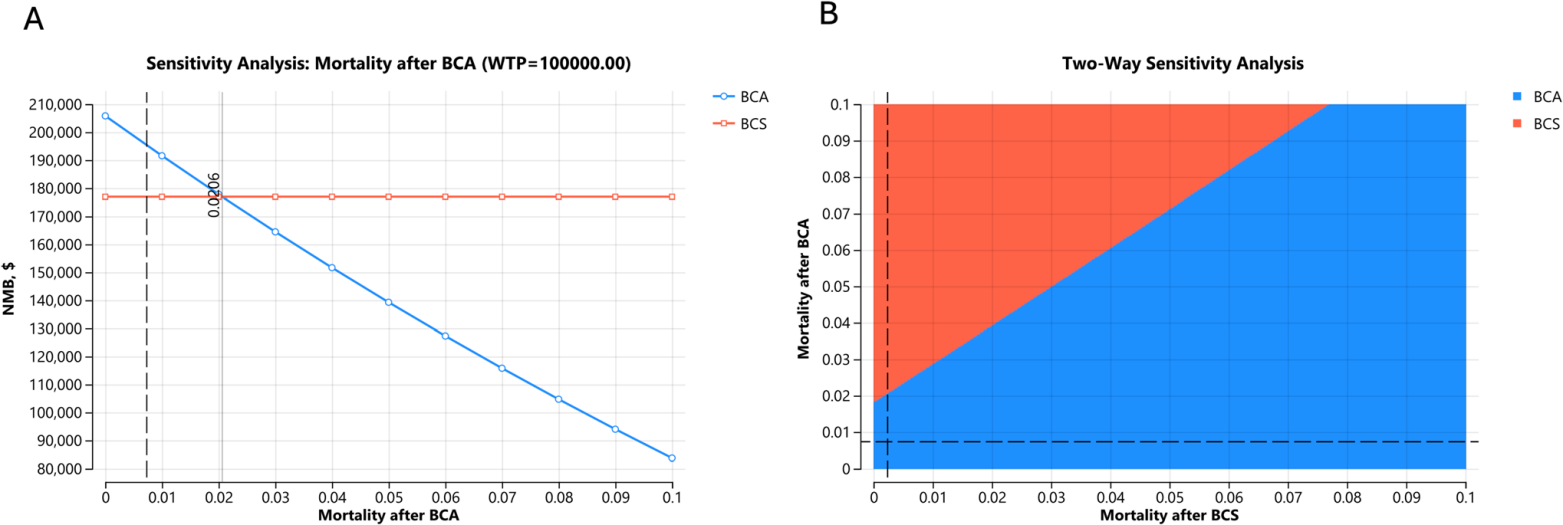
One- (**A**) and Two-Way (**B**) sensitivity analysis varying the mortality after BCA and BCS

When varying the annual mortality associated with breast cancer after BCA from 0 to 10%, BCS became the better strategy when the annual mortality after BCA was above 2.1% per year, equivalent to a 5-year cancer survival after BCA $<$ 90.0% (ICE3 result: 96.2% at 5 years) (Fig. 3A). Two-way sensitivity analysis, varying mortality after both BCS and BCA, showed that BCS became the more cost-effective strategy if the BCS mortality was more than 1.9% lower than that after BCA (Fig. 3B).

**Fig. 4:**
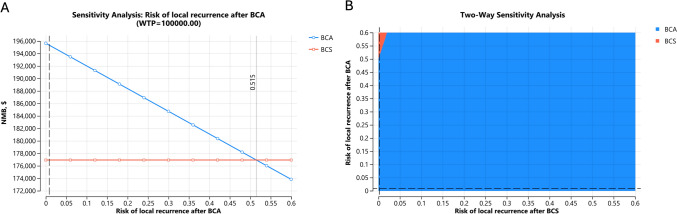
One- (**A**) and Two-Way (**B**) sensitivity analysis varying the risk of local recurrence after BCA and BCS

**Fig. 5:**
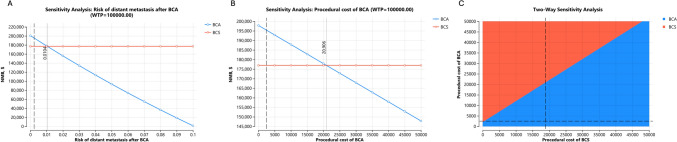
**A** One-way sensitivity varying the distant metastasis risk after BCA. One- (**B**) and Two-Way (**C**) sensitivity analysis varying the procedure costs of BCA and BCS

BCA was the optimal strategy when its local recurrence risk was < 51.5% per year (Fig. 4A) For reference, the IBTR reported in ICE3 was 4.3% at 5-year follow-up. Varying the IBTR of BCS from 0 to 10% per year did not change the conclusion, with a base case annual risk of 0.2%. Two-way sensitivity analysis showed BCA to be the more cost-effective strategy if its IBTR was less than 50% higher than that of BCS per year (Fig. 4B). The corresponding threshold value for distant recurrence per year was 1.04%, below which BCA was more cost-effective (Fig. 5A). Only 2 patients (1.03%) developed distant metastasis in ICE3 during a mean follow-up of 54 months (annual risk of 0.7%). Subset analysis assuming equal distant recurrence risks between the two modalities showed similar results, with BCA being the more cost-effective strategy.

**Fig. 6:**
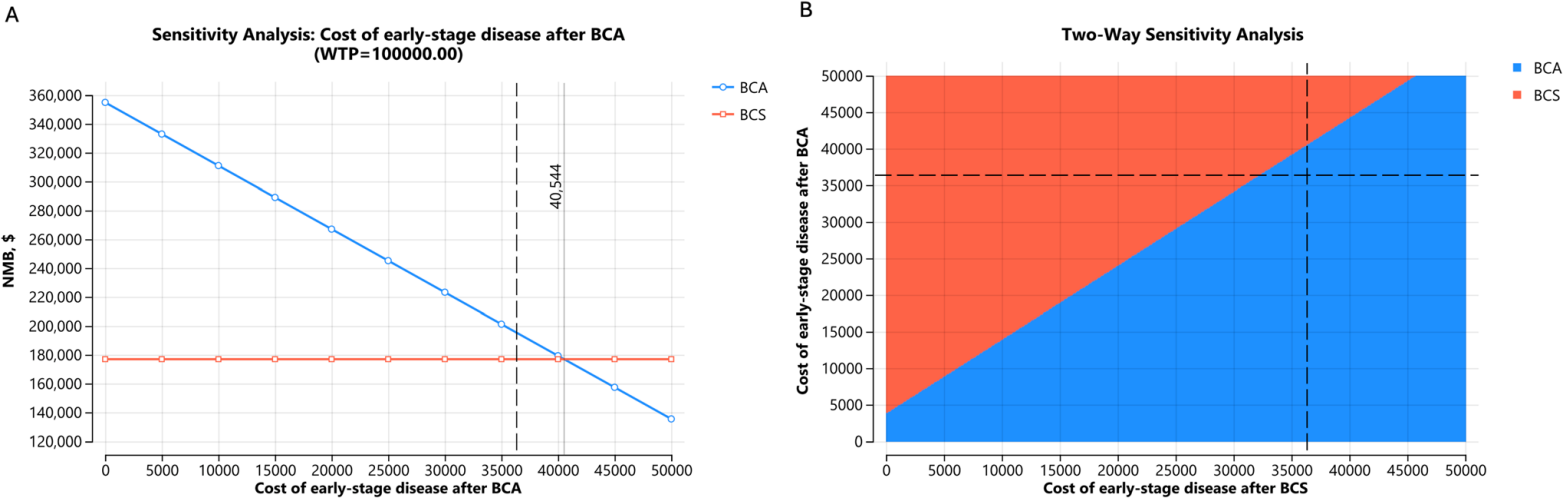
One- (**A**) and Two-Way (**B**) sensitivity analysis varying the follow-up cost of early-stage disease after BCA and BCS

Regarding treatment costs, BCS became the more cost-effective strategy if the cost of BCA was greater than $20,906 or if BCS was less costly than BCA (Fig. 5B and C). Varying the costs of subsequent follow-up medical care for surveillance, BCS was more cost-effective if the subsequent care after BCA was $4000 higher per year than BCS (Fig. 6A and B). Of note, the 2024 Medicare reimbursement rate for breast MRI with and without contrast was $351.85 (including both professional and technical fees). Varying the complication risks of both treatment strategies up to 50% did not change the conclusion. Varying the quality-of-life estimates associated with local and distant recurrence did not change the conclusion, although the utility value of IBTR affected BCA to a greater extent. When the disutility from undergoing BCA exceeded 20% (base case 3.5%), BCS (base case 9.5%) became more cost-effective.

Analyses performed using a WTP of $50,000/QALY were included in Appendix A, which showed unaffected base case and probabilistic sensitivity analyses given the lower cost and higher effectiveness of BCA. Decreasing the WTP liberalized most of the threshold values for BCA in deterministic sensitivity analyses, and conclusions remained similar.

## Discussion

This modeling-based analysis showed US-guided BCA to be more cost-effective than BCS over a 5-year horizon from a healthcare payer’s perspective, yielding comparable outcomes (0.01–0.09 QALY higher than BCS) at a lower cost ($17,000–$33,000 savings per patient). The conclusion remained so until the IBTR after BCA was higher than 51.5% per year, the distant recurrence risk higher than 1.0% per year, or the annual cancer mortality higher than 2.1%. BCA was no longer cost-effective if its subsequent annual surveillance cost was more than $4000 higher than after BCS. BCA was the more cost-effective strategy provided its total procedural cost was lower than that of BCS its long-term outcomes were similar. Immediate periprocedural complication risks and utility values associated with recurrence did not affect the conclusion.

Patient selection is crucial for US-guided BCA in breast cancer patients. Several early studies demonstrated the importance of targeting invasive ductal carcinomas with minimal associated ductal carcinoma in situ (DCIS), as the latter is often sonographically occult. One of the first clinical studies on US-guided cryoablation by Pfleiderer et al. included patients who underwent surgical resection 1–5 days after ablation. No remaining invasive carcinoma was seen in surgical specimens of tumors ≤ 1.5 cm, but sonographically occult DCIS were found adjacent to 40% of the ablated lesions upon histologic review [17]. The first multi-center trial, ACOSOG (Alliance) Z1072 enrolled 86 patients with invasive ductal carcinoma ≤ 2 cm and < 25% intraductal component. All patients underwent resection within 28 days after cryoablation. Complete pathologic response was only 75.8% (66/87), but this was largely due to synchronous lesions away from the ablation zone; complete necrosis in the ablation zone was noted in 19 of 21 cases. Viable cancer was found only at the edge of the cryoablation zone in the remaining two cases. Success rate was 92% after excluding multifocal disease and 100% for tumors ≤ 1 cm [18]. As a result of these findings, an exclusion criterion in ICE3 was an intraductal component of > 25% on core biopsy [9].

US-guided BCA is well-tolerated, with complication rates comparable to image guided core biopsy. No severe adverse events were reported in the ICE3 trial, and only five moderate adverse events occurred. All complications reported resolved with conservative management [19]. This is in line with other prospective and retrospective studies on US-guided BCA for either malignancy or fibroadenoma, which also demonstrated favorable safety profiles [20, 21]. Khan et al. [13] showed better physical and sexual well-being and cosmetic satisfaction in the cryoablation group based on BREAST-Q survey results. A Japanese cohort study also demonstrated high levels of patient satisfaction and quality of life post-ablation during the 5-year follow-up period [22].

BCA was the cost-effective strategy for small, low-risk breast cancers in this study largely because of its lower procedural costs. Compared to BCS, which generally involves operating room time and general anesthesia, US-guided BCA can be performed in an office-based setting under local anesthesia only [23]. Khan et al. found significantly lower direct and indirect costs and less financial burden for breast cancer patients (similar selection criteria compared to ICE3) undergoing BCA compared to BCS. Avoiding the operating room and general anesthesia was the primary driver of the lower procedural costs reported. Additional benefits of cryoablation may include less anxiety for patients undergoing the procedure in a clinic setting and improving operating room availability for more complex cases. Cryoablation patients also had higher financial toxicity scores (with higher scores indicating more favorable outcomes), suggesting a lower economic burden associated with their cancer care [13].

There are several limitations of the current study, which relies heavily on the ICE3 trial. The patient population enrolled in ICE3 was highly selective and in a non-randomized setting. Only patients 60 years and older with low-risk cancer were included, which was defined as hormonal receptor positivity and HER2 negativity. The size limit was 1.5 cm to allow for complete tumor ablation by a single cryoablation probe. However, most surgical literature on BCS uses an upper limit of 2.0 cm for tumors, consistent with stage I based on TNM staging [24]. The EBCTCG study also used 2.0 cm as the size threshold. While the authors performed subgroup survival and recurrent analyses based on size and low-risk features, the results were not specific to patients possessing both features. Therefore, the more conservative estimates of mortality and recurrence risks were used in the current study, and sensitivity analyses with wide ranges were performed to compensate for this limitation. Beyond the selectivity of its cohort, the ICE3 trial has a comparatively small sample size and shorter follow-up compared to the meta-analysis used for BCS herein, primarily owing to the novelty of BCA. The model therefore incorporated a time horizon of 5 years, mirroring the follow-up period of ICE3 trial, but the longer-term cost-effectiveness of BCA relative to BCS is unknown. Another limitation is that treatment modality may affect pre- and post-procedural costs. While the procedural costs of breast cryoablation were lower, pre-procedural work-up and post-treatment follow-up might be more costly compared to BCS. For example, some providers may use MRI to exclude multifocal disease or significant intraductal component prior to cryoablation, as done in the Z1072 trial [18]. More frequent or additional imaging may also be performed after cryoablation to ensure adequate treatment, which was seen in some early studies [25]. These additional costs before and after BCA may alter the cost-effectiveness of the modality, which was assessed via sensitivity analyses above. With increased experience and knowledge of expected post-ablation imaging findings, the follow-up cost after BCA will likely decrease over time. Finally, this analysis was limited by its use of United States-specific cost parameters and accepted WTP threshold. While cost parameters in other countries may vary, decreasing the WTP to $50,000 notably did not change the conclusion (Appendix A).

Future studies will make a cost-effectiveness analysis between BCA and BCS more robust. In addition to studies reporting long-term BCA outcomes, a randomized controlled trial comparing BCA and BCS would minimize selection bias between the two modalities. Including younger patients and patients higher-risk breast cancers in future BCA studies will also improve the generalizability of the results. In addition, employing real-world costs and quality of life data in multiple countries would help elucidate the cost-effectiveness of BCA and BCS for different health care systems.

In conclusion, US-guided cryoablation appears to be a cost-effective modality to treat early-stage, low-risk, sonographically visible breast cancer, with similar outcomes and lower costs compared to breast-conserving surgery. However, longer-term studies after cryoablation are needed to validate these findings.

## Supplementary Information

Below is the link to the electronic supplementary material.

Supplementary file1 (DOCX 17 KB)

Supplementary file2 (TIFF 87897 KB)
