# Supplementary material for: Cryoablation Versus Breast-Conserving Surgery for Early-Stage, Low-Risk Breast Cancer ≤ 1.5 cm: A Cost-Effectiveness Analysis
Source: Cardiovasc Intervent Radiol. 2025 Nov 18;49(2):313–21. doi: 10.1007/s00270-025-04269-3 (PMC12868015; doi:10.1007/s00270-025-04269-3)
Supplement: Supplementary file 1 — Supplementary file1 (DOCX 17 KB) [file 270_2025_4269_MOESM1_ESM.docx]

Appendix A - Analyses for WTP of $50,000/QALY

Base case analysis after BCS for low-risk, 1 – 20 mm breast cancers did not change with the lower WTP, and BCA remained the dominant strategy given its lower cost and higher effectiveness. Similarly, BCA was the better strategy in both scenarios among in greater than 99% of the 10,000 iterations in probabilistic sensitivity analyses.

Costs of local recurrences, breast cancer mortality, and recurrence risks (both local and distant) remained the key parameters in deterministic sensitivity analyses.

When varying the annual mortality associated with breast cancer after BCA, BCS became the better strategy when the annual mortality after BCA was above 2.9% per year or 5-year equivalent cancer survival rate of 86.3% (ICE3 result: 96.2% at 5 years). Two-way sensitivity analysis, varying mortality after both BCS and BCA, showed that BCS became the more cost-effective strategy if the BCS mortality was at least 2.8% lower than that after BCA.

BCA was the optimal strategy when its local recurrence risk was < 50.4% per year. Varying the IBTR of BCS from 0 – 10% per year did not change the conclusion, with a base case annual risk of 0.2%. Two-way sensitivity analysis showed BCA to be the more cost-effective strategy if its IBTR was less than 50% higher than that of BCS per year. The corresponding threshold value for distant recurrence per year was 1.1%, below which BCA was more cost-effective. Subset analysis assuming equal distant recurrence risks between the two modalities showed similar results, with BCA being the more cost-effective strategy.

BCS became the more cost-effective strategy if the cost of BCA was greater than $20,540 or if BCS was less costly than BCA. Varying the costs of subsequent follow-up medical care for surveillance, BCS was more cost-effective if the subsequent care after BCA was $4,000 higher per year than BCS. Varying the complication risks of both treatment strategies up to 50% did not change the conclusion. Varying the quality-of-life estimates associated with local and distant recurrence did not change the conclusion, although the utility value of IBTR affected BCA to a greater extent. When the disutility from undergoing BCA exceeded 39.8% (base case 3.5%), BCS (base case 9.5%) became more cost-effective.
